# 

**Published:** 1900-12-29

**Authors:** 


### NOTICE TO CORRESPONDENTS.

All MSS., Letters, Books for Review, and other matters intended for the Editor should be addressed THE EDITOR, "THE HOSPITAL," THE HOSPITAL BUILDING, 28 & 29 SOUTHAMPTON STREET, STRAND, W.C.

The Editor cannot undertake to return rejected MS., even when accompanied by stamped directed envelope.

**Notice to Advertisers and Subscribers.**

All Advertisements, Orders for Copies of THE HOSPITAL, and Business Communications should be addressed to **THE MANAGER (Not to the Editor)**, THE HOSPITAL, 28 & 29 Southampton Street, Strand, London, W.C.

The Cost of Subscription, post free, is as follows:—Per week, 2½d.; per quarter, 2s. 8d.; per half-year, 5s. 3d.; per annum, 10s. 6d. Foreign:—Per annum, 15s. 2d., payable, in all cases, in advance.

**NOTICE.—Vol. XXVIII. of "The Hospital" (April to September, 1900), in handsome cloth binding, is now on sale at the Office of this Journal, price, 7s. 6d. Cases for binding the Numbers contained in this Volume 1s. 6d. Index to Vol. XXVIII., 2½d., post free.**

**The Monthly Part for January will be ready on January 1st. Price 6d., by post 8d.**

### BOOKS RECEIVED.

Livingstone College.

"Seventh Annual Report of Livingstone College."

Cassell and Co.

"Plague: How to Recognise, Prevent, and Treat Plague." By Ja Cantlie, M.A., M.B.Aberd., F.R.C.S.Eng., D.P.H.Lon

### SCRAPS AND GLEANINGS.

Lord Penrhyn has made a donation of £100 to the Carnarvonshire and Anglesey Infirmary.

Her Majesty the Queen has made a present of 65 lb. of cast linen to the Cancer Hospital (Free), Fulham Road.

In aid of the Aberdeen Royal Infirmary, the parishes of Mildrum and New Pitsligo have organised house-to-house collections, which have realised £60 odd.

The shilling collection organised by the Cardiff *Western Mail* on behalf of the Cardiff Infirmary is finding many responses. In eight days the total was 8,251 shillings.

During the past year 190 patients have been admitted to the Derbyshire Children's Hospital, which is an increase of 42 on the previous year. There were 1,629 out patients.

A further addition to the Aldershot Infectious Hospital is to be put in hand at once at an approximate cost of £3,000. These buildings will include a new ward block, administrative block, a discharging ward, &c.

On the 31st inst. the Guildhall Banquet to the children of the Ragged School Union will be repeated under the auspices of Sir William Treloar. To the cripples compelled to remain at home 5,000 hampers, valued at 5s. each, will be distributed.

The directors of the Cardiff *Western Mail* have contributed 500 guineas to the Mayor's Cardiff Infirmary Fund. Strenuous efforts are being made to raise a large sum for the Cardiff Infirmary through the agency of this fund, and it is hoped that the principals of the Cardiff schools may co-operate in arranging concerts and entertainments on its behalf.

The total ordinary income of the Dudley Guest Hospital during the past year was £3,487. The private subscriptions amounted to £499 odd, as against £506 the previous year, and it was stated that for the past ten years the subscriptions from this source have shown a continuous decrease. The workmen's subscriptions amounted to £767. The expenditure for the year amounted to £3,614, so that, carrying forward the adverse balance from last year, there is a debit balance against the hospital of £246.

The Hon. Sec. of the Western Counties Convalescent Home is appealing for funds to carry on the Convalescent Home for sick and wounded soldiers at Combe Down. During the nine months which have passed since its establishment 77 soldiers have been received. It is desired to keep the home open till Midsummer, 1902, and to do this £800 must be raised in addition to the £540 now in hand, being the balance over from the original sum collected of £1,500, which was raised chiefly in the counties of Devon, Somerset, and Gloucester.

### The Student of Medicine.

### APPOINTMENTS.

**W. J. Barclay,** M.B., Ch.B., appointed Resident Medical Assistant at the Dundee Infirmary. **C. Osmond Bodman,** M.B., B.S.Durh., M.R.C.S.Eng., L.R.C.P.Lond., appointed Resident Medical Officer to the Royal Hospital for Children and Women, Waterloo Bridge Road. **W. G. H. Bradford,** B.A., M.B., B.C.Cantab., appointed Medical Officer and Public Vaccinator for the Debenham District of the Bosmere and Claydon Union. **Elph. J. De Sylva,** B.A., L.R.C.P.Edin., L.R.C.S.Edin., L.F.P.Glasg., appointed Medical Officer to the Parish of Lingwall, Whiteness and Weisdale, Shetland Islands. **Robert Dey,** M.B.Syd., appointed Government Medical Officer and Vaccinator at Wilcannia, New South Wales. **Geo. H. J. Dinsmore,** M.B., C.M., appointed Medical Officer for the Eccles Parish, Berwickshire. **C. A. Ensor,** M.R.C.S., L.R.C.P., appointed District Medical Officer and Medical Officer of the Workhouse of the Tisbury Union. **Frederick Henry Flack,** M.B., Ch.B. Vict. Univ., appointed Junior House-Surgeon to the Blackburn and East Lancashire Infirmary. **John K. Freyer,** L.R.C.S., L.K.Q.C.P.Irel., appointed Government Medical Officer and Vaccinator at Campbelltown, New South Wales. **Victor E. Gostling,** M.R.C.S., L.R.C.P., appointed House-Physician to the Derbyshire Royal Infirmary. **Edward Harrison,** M.A., M.D.Cantab., F.R.C.S.Eng., appointed Honorary Surgeon to the Hull Royal Infirmary. **Malcolm L. Hepburn,** M.D., B.S., F.R.C.S., appointed Surgeon to the Lowestoft Hospital. **James S. Hunt,** M.R.C.S.Eng., L.R.C.P.Edin., appointed Superintendent of the Institution for Inebriates at the Dunwich Benevolent Asylum, Queensland. **Harold J. Hutchens,** appointed Medical Officer of Health to the Joint Board for the Prevention of Epidemic Diseases, Brisbane. **Caleb Joyce,** M.B.Melb., appointed Health Officer to the Beaconsfield Board of Health, Tasmania. **J. Ellis Kilvert,** M.R.C.S., L.R.C.P., appointed House-Surgeon to the Derbyshire Royal Infirmary. **Henry A. Leschen,** M.B., Ch.M.Edin., appointed Medical Superintendent at the Whitby Falls Lunatic Asylum, West Australia. **Herbert C. McDouall,** M.R.C.S., L.R.C.P.Lond., appointed Senior Medical Officer at the Hospital for Insane, Callan Park, New South Wales. **William James Mackie,** M.D.Brux., L.R.C.S., L.K.Q.C.P.Irel., appointed Medical Officer of the Lunatic Asylum, Nelson, New Zealand. **W. P. Montgomery,** M.A.Oxon., M.B. and B.S.Lond., F.R.C.S., appointed Assistant Surgeon to the Manchester Children's Hospital. **G. Newman,** M.D.Edin., D.P.H.Camb., appointed Medical Officer of Health for the Borough of Finsbury. **George Rice,** M.D.Durh., appointed Honorary Physician to the Derbyshire Royal Infirmary. **Arthur Robinson,** M.D.Edin., appointed Professor of Anatomy at King's College, London. **John C. Shaw,** M.R.C.S., L.R.C.P., appointed Surgeon to the Royal Ear Hospital, Soho. **J. Acton Southern,** M.R.C.S., L.R.C.P., appointed Honorary Surgeon to the Derbyshire Royal Infirmary. **J. A. Turner,** M.B., C.M.Edin., D.P.H.Camb., appointed Executive Health Officer of Bombay.

